# Confinement‐Enhanced Multi‐Wavelength Photon Upconversion Based on Triplet–Triplet Annihilation in Nanostructured Glassy Polymers

**DOI:** 10.1002/advs.202415160

**Published:** 2025-02-14

**Authors:** Xueqian Hu, Luca Pollice, Alessandra Ronchi, Marco Roccanova, Michele Mauri, Davide Lardani, Dimitri Vanhecke, Angelo Monguzzi, Christoph Weder

**Affiliations:** ^1^ Adolphe Merkle Institute University of Fribourg Chemin des Verdiers 4 Fribourg 1700 Switzerland; ^2^ Dipartimento di Scienza dei Materiali Università degli Studi Milano‐Bicocca Via Roberto Cozzi 55 Milano 20125 Italy

**Keywords:** nanomaterials, polymers, solar harvesting, triplet‐triplet annihilation, upconversion

## Abstract

Sensitized triplet–triplet annihilation photon upconversion (sTTA‐UC) allows blue‐shifting non‐coherent low‐intensity light and is potentially useful in solar‐powered devices, bioimaging, 3D printing, and other applications. For technologically viable solar energy harvesting systems, solid materials that capture a large fraction of the solar spectrum and efficiently upconvert the absorbed energy must be developed. Here, it is shown that broadband‐to‐blue UC is possible in air‐tolerant, easy‐to‐access, nanostructured polymers comprising a rigid hydrophilic matrix and liquid nanodroplets with dimensions on the order of tens of nanometers. The droplets contain 9,10‐bis[(triisopropylsilyl)ethynyl] anthracene (TIPS‐Ac) as emitter/annihilator and palladium(II) octaethyl porphyrin (PdOEP) and palladium(II) meso‐tetraphenyl tetrabenzoporphine (PdTPBP) as sensitizers. The confinement of the three dyes in the liquid domains renders the various bimolecular energy transfer processes that are pivotal for the TIPS‐Ac's triplet sensitization highly efficient, and the simultaneous use of multiple light harvesters with triplet energy levels resonant with the emitter/annihilator increases the absorption bandwidth to ca. 150 nm. The UC process at low power densities is most efficient when both sensitizers are simultaneously excited, thanks to their confinement in the nanodroplets, which leads to an increase in the triplet density, and therefore TTA rate and yield, optimizing the use of the harvested energy.

## Graphical abstract

Nanostructured polymers that exhibit sensitized triplet–triplet annihilation (TTA) enable broadband‐to‐blue upconversion of low‐intensity light. The materials are comprised of a rigid matrix polymer and liquid nanodroplets containing an emitter and two sensitizers. The confinement of the dyes in the nanodomains makes the necessary energy transfer (ET) processes highly efficient, and the simultaneous use of two sensitizers leads to broad absorption.

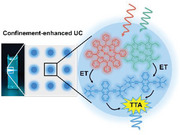

## 1 Introduction

Photon upconversion via sensitized triplet‐triplet annihilation (sTTA‐UC) permits transforming lower‐energy into higher‐energy electromagnetic radiation.^[^ ^1^ ^]^ sTTA‐UC involves the absorption of incident light by a sensitizer, intersystem crossing (ISC) between the sensitizer's singlet and triplet excited states, triplet‐triplet energy transfer (TTET) to an emitter/annihilator, and lastly triplet‐triplet annihilation (TTA) upon the encounter of two excited emitter molecules.^[^ ^2^ ^]^ The TTA step causes one of the excited emitter molecules to decay non‐radiatively to the ground state while the other is promoted to a singlet excited state from which delayed fluorescence occurs. Overall, this sequence formally combines two photons into one with higher energy than the absorbed ones.^[^ ^3^ ^]^ While sTTA‐UC was first reported 60 years ago by Parker and Hatchard,^[^ ^4^ ^]^ its exploitation in polymers, mainly driven by potential applications in solar power harvesting,^[^ ^5^ ^]^ bioimaging,^[^ ^6^ ^]^ photocatalysis,^[^ ^7^ ^]^ anticounterfeiting,^[^ ^8^ ^]^ 3D printing,^[^ ^9^ ^]^ and others,^[^ ^10^ ^]^ is a more recent development.^[^ ^11^ ^]^ Unlike two‐photon absorption,^[^ ^12^ ^]^ second‐harmonic generation,^[^ ^13^ ^]^ and upconversion with lanthanide‐based materials,^[^ ^14^ ^]^ the sTTA‐UC process can be used to blue‐shift non‐coherent, low‐power‐density radiation (<100 mW cm^−2^), and this renders the process potentially useful to harvest solar power.^[^ ^15^ ^]^ In the case of solar cells, the possibility to convert low‐energy photons below the semiconductor bandgap into higher‐energy photons would allow overcoming the Shockley‐Queisser limit; a detailed balance model shows that under ideal conditions, the maximum energy conversion of single‐junction bifacial solar cells can be raised by up to 47.6%.^[^ ^16^ ^]^ Similarly, the broad‐band upconversion of visible into blue light (<500 nm) would be extremely useful for photochemical reactions and photocatalytic water splitting,^[^ ^17^ ^]^ where high‐energy photons are required.^[^ ^18^ ^]^

To exploit sTTA‐UC in solar harvesting applications, efficient solid‐state upconverters are needed, which can be integrated with the actual harvesting device and exhibit broad‐band absorption so that incident light absorption is maximized.^[^ ^19^ ^]^ However, the best sensitizers developed for sTTA‐UC, notably metallated porphyrins, have narrow absorption bands and absorb only a small fraction of the incident photons.^[^ ^20^ ^]^ Efforts to address this problem by chemically modifying existing motifs are hindered since the relation between molecular structure, ISC, and electronic energy levels is complicated,^[^ ^21^ ^]^ which makes the rational modification of known sensitizers challenging. Another possibility is to employ auxiliary organic dyes or inorganic nanocrystals with broad‐band absorption, either as a sensitizer or to sensitize the sensitizer, from where the energy can be further transferred to the emitter triplets.^[^ ^22^ ^]^ A third approach to broaden the absorption of an sTTA‐UC system is to combine multiple sensitizers that exhibit different absorption bands but can transfer their energy to the same emitter. This strategy was first reported by Baluschev et al., who combined the two sensitizers meso‐tetraphenyl‐tetrabenzoporphine palladium (PdPh_4_TBP) and tetrakis‐meso‐(3,5‐dimethoxyphenyl) tetranaphthalo[2,3] porphyrin palladium (PdPh_4_MeO_8_TNP) with 5,6,11,12‐tetraphenylnaphthacene (rubrene) as the emitter in degassed toluene.^[^ ^23^ ^]^ The study demonstrated that combining two sensitizers can increase the excitation window and the upconverted emission intensity. A recent study by Monguzzi et al., who combined a family of asymmetric naphthobenzoporphyrins as complementary sensitizers with perylene in tetrahydrofuran, also documents an enhancement in light harvesting and sTTA‐UC for a multi‐sensitizer system in solution.^[^ ^21a^ ^]^ A first solid multi‐sensitizer sTTA‐UC system was recently reported by Kim and co‐workers, who observed a pronounced enhancement in UC fluorescence under low‐power excitation in polyurethane films containing platinum(II) octaethylporphyrin (PtOEP) and PdTPBP as sensitizers and perylene as an emitter.^[^ ^24^ ^]^ However, the chromophore concentrations were kept low to avoid aggregation, and this limited the material's absorbance. Moreover, the viscoelastic nature of the polymeric host renders the mobility of the dye molecules and, thereby, the external UC efficiency of such blends is low.^[^ ^3^, ^25^ ^]^ More importantly, most reports on multi‐sensitizer UC systems focused on expanding excitation windows, while a more comprehensive understanding of the possible synergistic effect of multiple sensitizers has yet to be developed.

**Figure 1:**
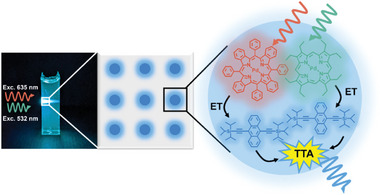
Schematic of the broadband photon upconverting materials developed. The nanostructured polymer matrix consists of a solid polymer, while the liquid nanodomains contain PdOEP and PdTPBP as sensitizers and TIPS‐Ac as emitters. The photograph was taken under simultaneous continuous‐wave excitation at 532 and 635 nm.

Here, we report multi‐wavelength sTTA‐UC in air‐tolerant solid materials that were accessed by incorporating two sensitizers that simultaneously activate one emitter into nanostructured polymers that are comprised of a rigid matrix and liquid nanodroplets with typical dimensions on the order of tens of nanometers.^[^ ^26^ ^]^ The continuous phase consists of a solid, hydrophilic, cross‐linked polymer matrix that contains liquid, hydrophobic nanodroplets in which the upconverting dye molecules are dissolved (**Figure** **1**). Such nanostructured multi‐sensitizer materials represent a flexible design approach to access upconverting materials that allow broadband light harvesting. The confinement of the three dyes in the liquid domains renders the various bimolecular energy transfer processes that are pivotal for the TIPS‐Ac's triplet sensitization highly efficient, and the simultaneous use of multiple light harvesters with triplet energy levels resonant with the emitter/annihilator increases the absorption bandwidth to ca. 150 nm. The UC process at low power densities is most efficient if both sensitizers are simultaneously excited, thanks to the confinement of light harvesters in the same nanodroplets^[^ ^27^ ^]^ that allows an efficient local increase in the triplet density, which maximizes the TTA rate and yield.

## 2 Design and Fabrication of the Nanostructured Upconverting Polymers

**Figure 2:**
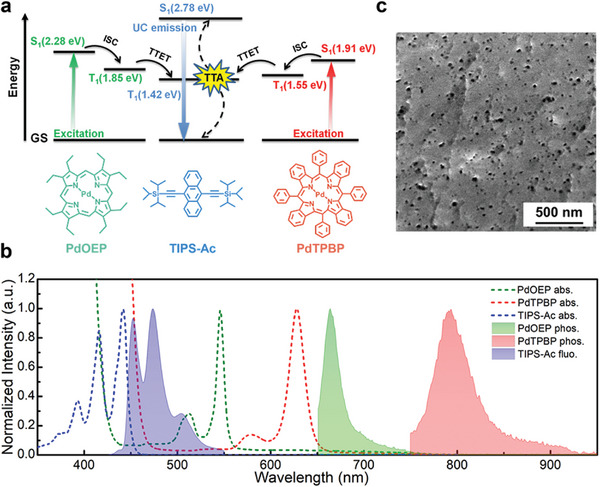
a) Jablonski diagram and schematic of the sTTA‐UC process at play in the dual‐sensitizer upconverting materials based on palladium(II) octaethyl porphyrin (PdOEP) and palladium(II) meso‐tetraphenyl tetrabenzoporphine (PdTPBP) as sensitizers and 9,10‐bis [(triisopropylsilyl)ethynyl] anthracene (TIPS‐Ac) as emitter/annihilator. ISC: intersystem crossing; TTET: triplet‐triplet energy transfer; GS: ground state; S_1_ and T_1_: first singlet excited and first triplet excited state of the corresponding molecule. b) Normalized absorption (dashed) and photoluminescence (PL) spectra (solid) of PdOEP (200 µm), PdTPBP (100 µm), and TIPS‐Ac (20 mm) in butyl benzoate (BuBz). c) Scanning electron microscopy (SEM) image of a multi‐wavelength upconverting nanostructured polymer sample fractured with liquid nitrogen. The image reveals a continuous polymer phase featuring nanosized pores with feature sizes of <50 nm.

Ideal sensitizers for light‐harvesting in sTTA‐UC schemes should possess a high molar extinction coefficient, high ISC yield, and triplet energy that is slightly above the one of the emitter. The fluorescence quantum yield of the emitter should be high and its dark triplets should be long‐lived to maximize the TTA probability.^[^ ^28^ ^]^ Both the sensitizer and the emitter have to be stable under operating irradiation conditions, and the overlap between the sensitizer's absorption and the emitter's upconverted emission spectra should be small to limit the energy back transfer and reabsorption.^[^ ^29^ ^]^ The red‐to‐blue upconverting sensitizer/emitter pair palladium(II) meso‐tetraphenyl tetrabenzoporphine (PdTPBP) and 9,10‐bis[(triisopropylsilyl)ethynyl] anthracene (TIPS‐Ac) (PdTPBP:TIPS‐Ac, **Figure** **2**a) meets these criteria and was previously reported to display a sTTA‐UC efficiency of 27% in degassed toluene solution.^[^ ^30^ ^]^ The combination of TIPS‐Ac and the well‐known palladium(II) octaethyl porphyrin (PdOEP) sensitizer (PdOEP TIPS‐Ac, Figure 2a) should allow green‐to‐blue UC, but unlike the combination of a similar sensitizer (platinum(II) octaethyl porphyrin) with TIPS‐Ac,^[^ ^31^ ^]^ the PdOEP:TIPS‐Ac pair remains, to our best knowledge, unexplored. The Jablonski diagram in Figure 2a summarizes the pertinent energy levels of PdTPBP, PdOEP, and TIPS‐AC and reflects that TTET from both sensitizers to TIPS‐Ac should be possible. The UV–vis absorption and emission spectra of dilute solutions of the three dyes in butyl benzoate (BuBz, the solvent used to fabricate the nanostructured polymers) are shown in Figure 2b. The absorption spectra reveal that the targeted materials should exhibit an exploitable absorption range from ca. 500 to 650 nm, with maxima at 546 and 628 nm, corresponding to green‐to‐blue and red‐to‐blue upconversion, as TIPS‐Ac displays a fluorescence spectrum ranging from ca. 450 to 550 nm. The absorption spectra also show that the PdOEP and PdTPBP Soret bands slightly overlap with the TIPS‐Ac emission spectrum at wavelengths below 450 nm, which may lead to (minor) back transfer from TIPS‐Ac to the sensitizers.

The nanostructured host material used here comprises a solid, cross‐linked matrix polymer that is formed by the free‐radical polymerization of 2‐hydroxyethyl methacrylate, methacrylic acid, and triethylene glycol dimethacrylate, as well as ca. 10 wt% of the hydrophobic solvent BuBz, which forms the liquid nanodroplets.^[^ ^26^, ^32^ ^]^ The host material further contains triethylene glycol (15 wt%) as a plasticizer, cetyltrimethylammonium chloride (5 wt%) as a surfactant, and of course, the upconverting dyes, which dissolve in the BuBz nanodroplets (Experimental Section). All samples were prepared by redox‐initiated polymerizations of non‐degassed reaction mixtures in sealed glass cuvettes with a path length of 1 cm. After the polymerizations were complete, the upconverting materials were removed from the cuvettes by breaking the glass and were handled and measured without further protection from environmental factors.

Adapting reported procedures,^[^ ^26b^ ^]^ we fabricated multi‐wavelength upconverting polymers containing PdOEP, PdTPBP, and TIPS‐Ac (PdOEP:PdTPBP:TIPS‐Ac), as well as the corresponding reference materials containing only PdOEP and TIPS‐Ac (PdOEP:TIPS‐Ac) or PdTPBP and TIPS‐Ac (PdTPBP:TIPS‐Ac). The reference polymers containing only one chromophore PdOEP, PdTPBP, or TIPS‐Ac were also prepared by the same procedures. While the composition of the host material was kept fixed, we varied the concentration of the dyes, as discussed below. Time‐domain NMR experiments on the PdOEP:PdTPBP:TIPS‐Ac sample show the existence of both mobile and rigid phases at room temperature (Figure S1, Supporting Information, rigid fraction 56% of the total), due to the presence of the plasticizers and of the BuBz organic solvent droplets in agreement with previous results (Experimental Section).^[^ ^26a^ ^]^ The Hahn Echo sequence analysis (Figure S1, Supporting Information) reveals a slow component with a relaxation time of ≈16 ms, which is in agreement with the typical mobility of viscous or confined liquids. Spin diffusion data reveal that the liquid domains, assumed to have a spherical shape, have a diameter of ≈15 nm, which is of the same magnitude as the dimensions of the porous features observed in scanning electron microscopy (SEM) images (Figure 2c).

## 3 Upconversion in Single‐Sensitizer Nanostructured Polymers

We investigated nanostructured upconverting polymers with only one sensitizer, i.e., PdOEP:TIPS‐Ac and PdTPBP:TIPS‐Ac, with the primary goals of establishing benchmarks and optimizing the concentrations of the dyes (Figure S2, Supporting Information). After establishing that 20 mm TIPS‐Ac solutions in BuBz are stable and show no signs of phase separation, this concentration was used for all materials (all concentrations are quoted relative to the volume of BuBz used in the synthesis) since a high emitter concentration is a prerequisite for efficient TTET and high annihilation yield. The concentration of the two sensitizers was varied between 100 and 1 mm to maximize their absorbance while limiting unavoidable energy back transfer from excited emitter molecules to the sensitizer (Figure S2, Supporting Information).^[^ ^33^ ^]^ Screening experiments show that the UC emission intensity upon continuous‐wave (CW) excitation with lasers at 532 or 635 nm for PdOEP:TIPS‐Ac and PdTPBP:TIPS‐Ac is maximum at PdOEP and PdTPBP concentrations of 200 and 100 µm, respectively (Figure S2a,b, Supporting Information). Notably, at these concentrations, at both wavelengths, >95% of the incident photons are absorbed over an optical path length of 1 cm (Figure S2, Supporting Information). Further increases in the sensitizer concentration lead to reduced UC emission, suggesting that energy backtransfer from the excited emitters to the sensitizer and/or reabsorption effects outweigh the benefits of increased absorbance.

**Figure 3:**
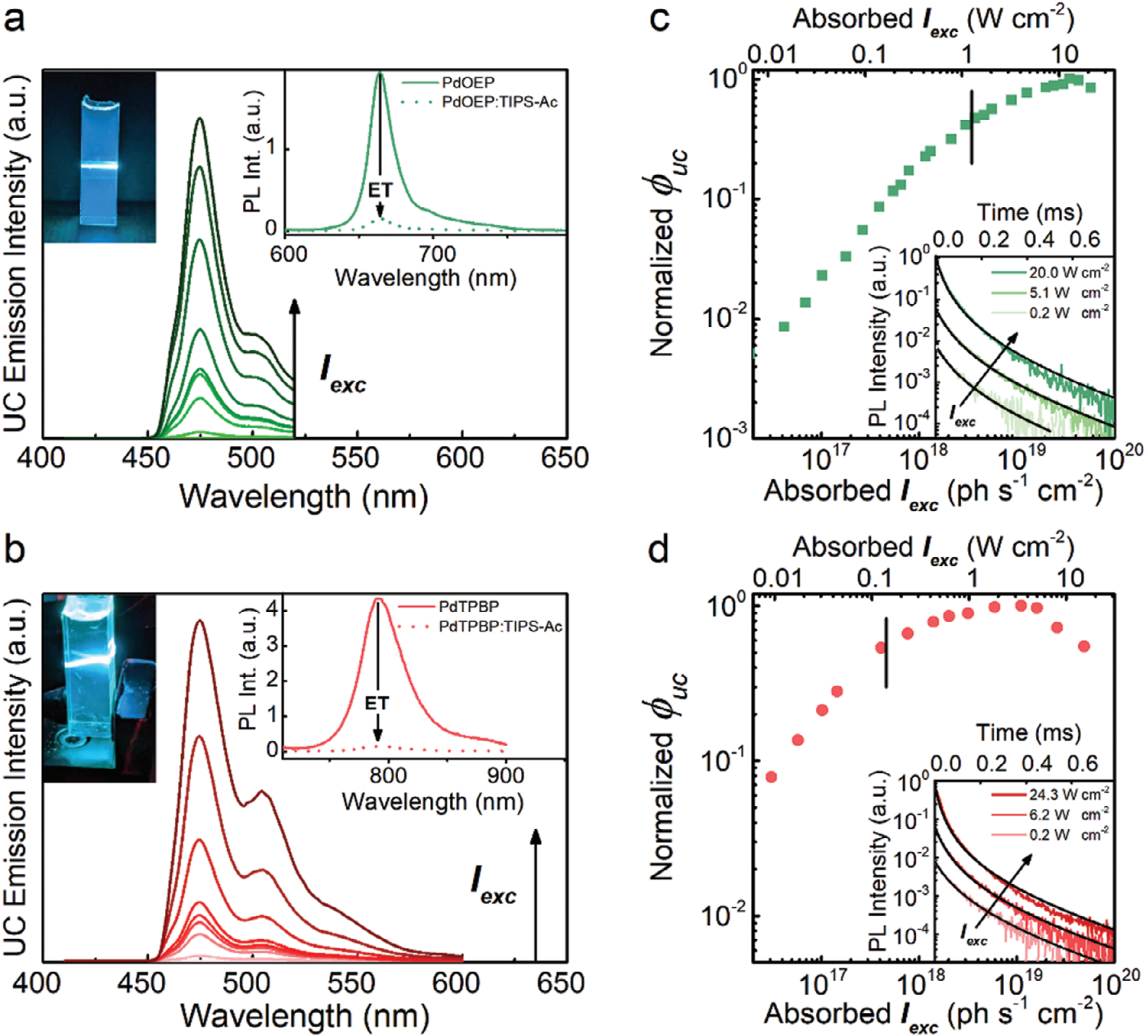
a,b) Upconversion (UC) emission spectra of the nanostructured upconverting polymers containing PdOEP (2 × 10^−5^ m, panel a) or PdTPBP (1 × 10^−5^ m, panel b) and the annihilator/emitter TIPS‐Ac (2 × 10^−3^ m) as a function of the absorbed excitation intensity *I*_exc_ at λ_ex_ = 532 nm (a) and λ_ex_ = 635 nm (b). The top right insets show the (a) PdOEP or (b) PdTPBP phosphorescence in nanostructured polymers without/with TIPS‐Ac; the intensity drop observed in the presence of the emitter demonstrates efficient triplet‐triplet energy transfer (TTET). The top left insets are digital pictures of the samples excited with (a) a green laser at 532 nm and (b) a red laser at 635 nm. c,d) Normalized UC yield *ϕ_uc_* measured as a function of *I_exc_* at (c) λ_ex_ = 532 nm and (d) λ_ex_ = 635 nm. The insets show the UC emission intensity decay with time as a function of *I_exc_* at 532 and 635 nm, respectively, fitted with multiexponential decay functions.

Based on these results, we fixed the concentration of PdOEP at 200 µm and of PdTPBP at 100 µm and investigated the green‐to‐blue and red‐to‐blue upconversion of PdOEP:TIPS‐Ac and PdTPBP:TIPS‐Ac nanostructured polymers in detail. Steady‐state photoluminescence spectra show that both materials exhibit upconverted blue emission that matches the TIPS‐Ac fluorescence when excited with continuous‐wave (CW) laser light at 532 nm or 635 nm, respectively (**Figure** **3**a,b). Efficient TTET is evident if one compares the phosphorescence intensity of the upconverting samples (*I_Ph_*) and reference materials without TIPS‐Ac $\left(I_{Ph}^{0}\right)$ (Insets of Figure 3a,b). The TTET yield $\phi_{ET}=\left(1-I_{Ph}/I_{Ph}^{0}\right)$ thus determined is 0.92 for PdOEP:TIPS‐Ac and 0.97 for PdTPBP:TIPS‐Ac. The fact that TTET is highly efficient is further demonstrated by time‐resolved analysis of the sensitizers’ residual phosphorescence. In both systems, dramatically shorter lifetimes are observed in the presence of TIPS‐Ac (Figure S3, Supporting Information). Plots of the normalized UC yield ϕ_*uc*_ as a function of the absorbed excitation intensity *I_exc_*, i.e., the incident intensity normalized by the material absorptance, show the typical smooth transition from a power‐dependent regime to a maximum efficiency regime as already observed for other nanostructured UC polymers, in which the confined TTA renders the UC process highly efficient (Figure 3c,d).^[^ ^26^, ^34^ ^]^ The occurrence of confined TTA in the present materials is confirmed by the UC emission decay kinetics, which is shown in the insets of Figure 3c,d. For both samples, the decay kinetics are independent of the *I_exc_*, which is a particular characteristic of the confined‐TTA regime.^[^ ^27^ ^]^ Notably, the UC emission decay rate *k_TTA_*, i.e., the reciprocal of the time τ_*uc*_ at which the UC emission intensity is reduced to 1/*e* of its initial value, is much larger than the spontaneous decay rate of TIPS‐Ac triplets of *k_T_* = 666 Hz (Figure S4, Supporting Information). In both compositions, the UC emission intensity drops in a few tenths of a microsecond, corresponding to a *k_TTA_* of ≈3 × 10^4^ Hz, independent of the excitation wavelength and the excitation intensity (Table S1, Supporting Information). This means that the TTA yield $\phi_{TTA}=\frac{k_{TTA}}{k_{TTA}+k_{T}}\;$ approaches unity for both single‐sensitizer materials, regardless of the excitation intensity because of the forced and fast annihilation of triplets entrapped in the nanodroplets.

## 4 Upconversion in Dual‐Sensitizer Upconverting Polymers

**Figure 4:**
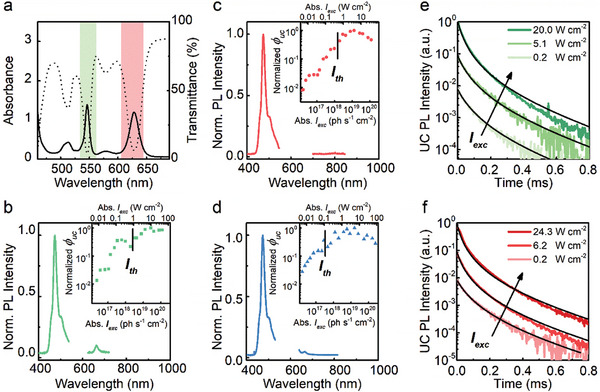
Optical characterization of the nanostructured upconverting polymer containing PdOEP (2 × 10^−5^ m), PdTPBP (1 × 10^−5^ m), and TIPS‐Ac (2 × 10^−3^ m). a) Absorption (solid) and transmission (dashed) spectra. b–d) UC emission spectra acquired with (b) λ_ex_ = 532 nm (2.2 W cm^−2^, top), (b) 635 nm (2 W cm^−2^, middle), and (d) simultaneous excitation at 532 nm (2.2 W cm^−2^) and 635 nm (2 W cm^−2^), respectively. The insets report the normalized UC yield *ϕ_uc_* as a function of the absorbed excitation power density *I_exc_*. Vertical lines mark the excitation intensity thresholds *I_th_*. e,f) Decay of the UC emission intensity under modulated excitation at (e) λ_ex_ = 532 nm or (f) λ_ex_ = 635 nm as a function of the excitation intensity.

After investigating the green‐to‐blue and the red‐to‐blue upconversion displayed by the single‐sensitizer materials, we combined the two sensitizers and the emitter in one nanostructured material (PdOEP:PdTPBP:TIPS‐Ac). Following the process detailed above, we fabricated a multi‐wavelength upconverting nanostructured polymer containing PdOEP (2 × 10^−5^ m), PdTPBP (1 × 10^−5^ m) and TIPS‐Ac (2 × 10^−3^ m). The absorption spectrum of the material (**Figure** **4**a) shows a superposition of the absorption bands of the three dyes, with maxima at 513, 546, 579, and 628 nm. The transmission spectrum shows that the multi‐sensitizer material absorbs at least 50% of the incident photons in a range from ca. 500 to 650 nm, and thus allows capturing a considerably larger fraction of light in the visible range than the corresponding single‐sensitizer materials.

As shown in Figure 4b–d, the upconverted emission spectra of the multi‐wavelength upconverting nanostructured polymer are identical and show the characteristic TIPS‐Ac emission under laser excitation at 532, 635 nm, or simultaneous double excitation at both wavelengths. The long‐term stability of the multi‐wavelength upconverting nanostructured polymer was evaluated by monitoring the intensity of the UC emission intensity under CW excitation for up to 27 days (Figure S5, Supporting Information). The material shows a stable UC performance both when sealed and released from the cuvettes, proving excellent shielding properties against atmospheric moisture.

The insets in Figure 4b‐d show the normalized UC yield ϕ_uc_ measured as a function of *I_exc_* under CW single‐wavelength or double‐wavelength excitation (Figure S6, Supporting Information). As for the single‐sensitizer nanostructured materials,^[^ ^26b,c^ ^]^ all plots display the characteristic smooth transition from a power‐dependent regime to a constant ϕ_uc_, where the sTTA‐UC efficiency is maximal. The efficiency reduction observed at very large excitation intensities can be ascribed to the saturation of the annihilator population and to singlet‐singlet annihilation.^[^ ^35^ ^]^ The transition from the power‐dependent to the maximum efficiency regime is marked by the excitation threshold intensity *I_th_*, at which ϕ_uc_ is half of its maximum. For the PdOEP:PdTPBP:TIPS‐Ac polymer under single excitation, we observe a *I_th_* of 2.6 × 10^18^ ph cm^−2^ s^−1^ (0.97 W cm^−2^) and 1.5 × 10^18^ ph cm^−2^ s^−1^ (0.47 W cm^−2^) for excitation at 532 and 635 nm, respectively, in agreement with previous results.^[^ ^26c^ ^]^ *I_th_* is reduced to 3.4 × 10^17^ ph cm^−2^ s^−1^ (0.12 W cm^−2^) when the excitation occurs with two lasers operating at 532 and 635 nm. In the maximum efficiency regime, we measure a ϕ_uc_ of 24 ± 4% and 16 ± 3% under excitation at 532 and 635 nm, respectively (Supporting Information, Section 3), in agreement with previous results and literature.^[^ ^3a^ ^]^

The UC emission decay kinetics in the multi‐wavelength nanostructured polymer under pulsed laser excitation at 532 or 635 nm are identical (Figure 4e,f), with a negligible dependence from the excitation intensity that further confirms the occurrence of the confined TTA regime, again with a close‐to‐unity ϕ_*TTA*_ regardless of the excitation intensity and with *k_TTA_* values comparable to those observed for the single‐sensitizer reference materials (Table S1, Supporting Information). This indicates the simultaneous presence of two sensitizers does not significantly affect the single UC processes. While no back‐transfer of triplets from TIPs‐Ac to the sensitizers is expected based on the triplet energies (Figure 2a),^[^ ^33^ ^]^ the TIPS‐Ac emission and the sensitizers’ absorption bands show some overlap (Figure 2b), suggesting that the back‐transfer of singlets from the emitter to the sensitizers may not be inconsequential. To investigate this, we recorded the fluorescence decay traces of nanostructured polymers containing various dye combinations under direct excitation of the TIPS‐Ac emitter at 405 nm (Figure S7, Supporting Information). The fluorescence lifetime decreases in the order τ_TIPS − Ac_ > τ_PdOEP: TIPS − Ac_ > τ_PdTPBP: TIPS − Ac_ > τ_PdOEP: PdTPBP: TIPS − Ac_, which is consistent with the increasing overlap integral between the TIPS‐Ac fluorescence and the sensitizers’ absorption in the same order. We calculated the singlet back‐transfer yield as $\phi_{bck}^{S}=1-\tau /\tau_{0}$ where τand τ_0_ are the TIPS‐Ac fluorescence lifetime measured in the presence and absence of the sensitizers. The $\phi_{bck}^{S}$ values thus determined are 0.13, 0.28, and 0.29 for the PdOEP:TIPS‐Ac, PdTPBP:TIPS‐Ac, and PdOEP:PdTPBP:TIPS‐Ac, respectively, and reflect that the singlet back transfer is moderate and mainly driven by the presence of PdTPBP.

To enable a more quantitative analysis of the UC performance of the multi‐wavelength upconverting material under simultaneous excitation of both sensitizers, we recorded the UC emission intensity of a nanostructured PdOEP:PdTPBP:TIPS‐Ac sample as a function of the absorbed photon flux under single and dual wavelength excitation in a side‐by‐side experiment. Considering that in all cases the TTET yield is close to 100%, the comparative analysis of the UC intensity as a function of injected triplets is highly informative if one recalls that in these nanostructured systems, confined TTA occurs and that the UC output is mainly determined by the probability of simultaneously creating two triplets in the same nanodroplet, thus activating TTA with ϕ_*TTA*_ ≈ 1.^[^ ^27^ ^]^ This probability is set by the number of light harvesters in each nanodroplet and by their absorption cross‐section at the excitation wavelength (Supporting Information, Section 2). Thus, by including two sensitizers that can harvest two photons at different frequencies under double‐wavelength excitation, the intrinsic probability of triplet creation in each nanodroplet is increased with respect to single‐wavelength excitation. This directly impacts the UC yield, which should be higher at low *I_exc_* because of the statistically enhanced probability of photon harvesting.

**Figure 5:**
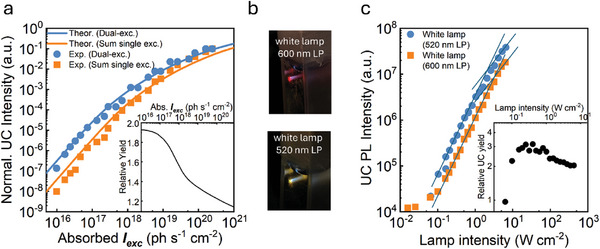
a) Normalized UC emission intensity of the nanostructured upconverting polymer containing PdOEP (2 × 10^−5^ m), PdTPBP (1 × 10^−5^ m), and TIPS‐Ac (2 × 10^−3^ m) under dual‐wavelength excitation at 532 and 635 nm (blue circles) and the sum of the UC emission intensities recorded under single‐wavelength excitation at 532 and 635 nm (orange squares) as a function of the absorbed power density *I_exc_*. The solid lines are the theoretically calculated emission intensity curves considering the probability of triplet exaction generation in each nanodroplet, the TTA yield as a function of the *I_exc_*, sensitizer concentration, and absorption cross‐section. Inset: relative UC yield under double‐wavelength excitation conditions versus single‐wavelength excitation conditions, calculated as the ratio between the two values as a function of *I_exc_*. b) Digital pictures of the upconverting nanostructured polymers under white lamp excitation using a 600 nm (top) long pass (LP) or 520 nm (bottom) LP optical filter at the maximum power employed. The blue upconverted emission is visible under the naked eye. c) UC emission intensity under CW broadband excitation with a white lamp using a 520 nm LP (blue circles) or 600 nm LP (orange squares) optical filter as a function of the incident intensity. Solid lines are fitted with quadratic and linear dependency on the excitation intensity, respectively. The inset reports the relative UC emission intensity (black circles).

**Figure** **5**a shows the normalized UC emission intensity that is measured when the nanostructured PdOEP:PdTPBP:TIPS‐Ac sample is simultaneously excited at 532 and 635 nm in comparison to a trace that shows the sum of the UC emission intensities recorded if the sample is separately excited at 532 and 635 nm. For this experiment, the laser beam intensities were adjusted to realize the same number of absorbed photons at the two wavelengths. Considering that the TTET is close to unity for both sensitizers, this means that each laser generates the same number of triplets in the system. Thus, the total number of triplets in the system under double excitation is exactly twice as high as the number of triplets generated by single excitation at either wavelength. The data show that at low power densities, the UC signal recorded upon multi‐wavelength excitation (Figure 5a, blue circles) is clearly higher than the sum of the UC emission intensities measured for the same material in separate single‐wavelength excitation experiments (Figure 5a, orange squares). Intriguingly, the experimental data can be perfectly reproduced by considering the effect of the presence of the second sensitizer in the activation of confined TTA in the nanostructured system (Figure 5a, solid lines, Supporting Information, Section 2).^[^ ^34^ ^]^ This consolidated modeling thus allows for the precise evaluation of the enhancement of the UC yield that results from dual‐wavelength excitation versus single‐wavelength excitation in a confined‐TTA system (Figure 5a, inset).

At low *I_exc_*, under the dual‐laser excitation, the UC yield is almost doubled with respect to the sum of two independent single‐laser activations, on account of the presence of two independent light harvesters that increases the intrinsic probability of populating a nanodroplet with multiple triplets (Supporting Information, Section 2). Accordingly, this gain progressively decreases when *I_exc_* is increased because the excitation photons flux becomes sufficiently large to guarantee the presence of multiple triplets in the same nanodroplet, even if only one sensitizer is excited. Indeed, as *I_th_* is reached or surpassed, most of the nanodroplets are efficiently populated by multiple triplets even under single‐wavelength excitation conditions, thus maximizing the TTA yield independently for the presence of a second light harvester.

The results clearly highlight the synergistic effect of two sensitizers at lower powers, thus suggesting potential applications for ultra‐low power uses where a broad absorption is mandatory, such as in solar‐powered devices. To demonstrate this, we performed an additional experiment, in which we excited the nanostructured PdOEP:PdTPBP:TIPS‐Ac sample with a low‐power, non‐coherent broadband white lamp equipped with either a 520 or 600 nm long‐pass optical filter (Supporting Information, Figures S9–S11, Supporting Information). Both filters eliminate the light that could directly excite the TIPS‐Ac emitter and allow a comparison of dual versus single sensitizer excitation with a non‐coherent source that mimics the sun. At the maximum available power, the UC blue emission can be seen by the naked eye (Figure 5b). Then the UC emission intensity was recorded as a function of the incident intensity of the lamp emission (Figure 5c). It is evident that excitation through the 520 nm bandpass filter, which causes the excitation of both sensitizers, leads to a significantly more intense emission and to a threshold of 1.3 W cm^−2^ that is half of the 2.6 W cm^−2^ observed using 600 nm long pass filter that allows the excitation of a single sensitizer using red light. The inset of Figure 5c shows the relative UC efficiency calculated as the ratio between the dual versus single UC emission intensities. The excitation lamp has a constant emission intensity in the spectral region of interest (Figure S9, Supporting Information), both sensitizers absorb 100% of the incident photons at the maximum of their absorption band, and the absorption bandwidths are similar. Thus, the relative UC yield has been normalized only to the energy of photons 547 and 635 nm to have a more realistic comparison as a function of the relative number of absorbed photons. In this condition, basically using the 520 nm filter, we inject twice the number of photons in the system than when using the 600 nm filter, so in the confined‐TTA regime, we expect a UC yield two times larger.^[^ ^27^, ^34^ ^]^ Interestingly, the data clearly demonstrate again the effective synergistic behaviour of the two sensitizers in activating the confined TTA at low power. When both sensitizers are excited, the UC yield increases progressively, showing an efficiency that is about three times higher than that measured upon sensitization in the presence of the 600 nm long‐pass filter, thus confirming the proposed modeling and the effectiveness of the multi‐sensitizer strategy. The UC experiments performed under broadband non‐coherent excitation allow estimating the potential effectiveness of the proposed system coupled to an ideal solar device with a bandgap at 520 nm, for example, a photocatalytic water splitting cell, powered by sunlight (Table S2, Supporting Information). Under AM.1.5 irradiation condition, the maximum UC yield regime can be reached only using concentrated sunlight, as previously proposed.^[^ ^36^ ^]^ In this case, considering the light‐harvesting ability of both sensitizers, the UC nanostructured polymer will absorb +38% photons with respect to the solar device. Considering the UC yield observed and using a back mirror to reflect the upconverted light into the photovoltaic device,^[^ ^37^ ^]^ this means that the light‐to‐electricity conversion can be improved by a factor up to +7%.

## 5 Conclusion

In conclusion, we designed and fabricated upconverting nanostructured polymers that exhibit broadband light absorption and sTTA‐based low‐power upconversion in ambient conditions. The combination of two sensitizers as light harvesters greatly expands the material's absorption window and allows the absorption of a three times larger portion of the incident solar spectrum compared to its counterparts containing only one sensitizer. This, in turn, leads to a higher concentration of available emitter triplets, enabling the system to reach the maximum efficiency regime with a halved excitation intensity threshold and producing 2–3 times more upconverted photons. The liquid nature of the upconverting phase in the nanostructured polymer makes the TTA process highly efficient, compensating for the emitter triplets’ low density under low‐power excitation. Moreover, we demonstrate that the two sensitizers work synergistically in the nanostructured upconverter by significantly increasing the UC yield at low powers and thus lowering the energy density required to maximize the UC light output. These results support that the nanostructured polymer system can act as a versatile platform to incorporate any dye pair or dye combination according to the desired application, overcoming the limitations typically affecting highly doped bulk materials.

## 6 Experimental Section

### Fabrication of Multi‐Wavelength Upconverting Nanostructured Polymers and Reference Materials

All chemicals were acquired from ABCR, Frontier Scientific, Sigma‐Aldrich, or TCI, and were used without further purification. The multi‐wavelength upconverting nanostructured polymers and the reference materials were fabricated by adapting previously reported procedures^[^ ^26b^ ^]^ without prior deoxygenation of the single components or their mixtures. To prepare the multi‐wavelength upconverting nanostructured polymer, methacrylic acid (665 mg), 2‐hydroxyethyl methacrylate (2.66 g), triethylene glycol (750 mg), cetyltrimethylammonium chloride (250 mg), and triethylene glycol dimethacrylate (175 mg) were mixed in a 20 mL glass vial before a butyl benzoate solution (500 mg) containing PdOEP (0.064 mg), PdTPBP (0.046 mg), and TIPS‐Ac (5.39 mg) was added. After adding a stir bar, the mixture was heated to 60 °C and stirred for 20 min until clear. The glass vial was removed from the heat, and a 30% aqueous hydrogen peroxide solution (10 mg) and subsequently 2‐mercaptoethanol (10 mg) were added. The vial was gently shaken, and the solution was rested for 1–2 min. Dimethylthiomethane (27 mg) was added, the mixture was briefly shaken, filtered through a syringe filter (0.2 µm PTFE), and transferred into a glass cuvette (external dimensions = 12.5 × 12.5 × 45 mm, internal width = 10 mm). The glass cuvette was sealed with a stopper and left to stand at room temperature overnight. The transparent, rigid material thus made was released from the cuvette for further use. Following similar procedures, the reference materials were prepared accordingly by omitting PdOEP, PdTPBP, or both sensitizers.

### Structural Characterization of Multi‐Wavelength Upconverting Nanostructured Polymers

Time domain ^1^H‐NMR experiments were conducted using a 0.5 T Brucker Minispec mq20 with a ^1^H Larmor frequency of 19.9 MHz. A static probe was used and the instrument was equipped with a BVT3000 temperature control unit operated with N_2_ (g). The temperature was controlled with an external thermometer that had an accuracy of 1 K and a precision of 0.1 K. To allow for thermal equilibration, the sample was kept within the magnet for approximately ten minutes prior to experimentation. For variable temperature measurements, the same thermal equilibration period was maintained before each data point acquisition. To determine the rigid phase, free induction decays (FIDs) were acquired following a pulsed mixed magic sandwich echo (MSE) sequence, encompassing 128 scans per sample. To calculate the domain size, calculations were carried out with FIDs obtained post an MSE refocused Goldman‐Shen sequence, utilizing the initial rate approximation for the rigid region. T2 relaxation times were assessed employing a standard Hahn Echo sequence, with receiver dead time set to 12.7 µs, phase switching time to 2.2 µs, and the 90° pulse length configured at 2.10 µs. Scanning electron microscopy (SEM), images were acquired on samples that had been fractured using N_2_ (l) and subsequently coated with a 4 nm layer of gold using a Cressington 208H (Liverpool, UK). SEM images were acquired using a Thermo Fischer (Waltham, MA, USA) SCIOS 2 field‐emission scanning electron microscope, operated at a beam voltage of 5 kV using the in‐lens secondary electron detector. The size and distribution of nanodroplets were calculated based on three different areas from SEM images using Fiji (ImageJ) software.

### Optical Characterization

UV–vis absorption spectra were recorded on a Shimadzu UV‐2401PC. Steady‐state photoluminescence experiments were carried out at room temperature with a N_2_‐cooled charge‐coupled device (Spex ≈2000) that was coupled to an imaging spectrometer (J‐Horiba Triax 190). Green‐to‐blue and red‐to‐blue upconversion spectra were recorded using focused, Nd:YAG diode pumped, frequency‐doubled Coherent Verdi TEM 00 CW lasers operated at 532 or 635 nm for excitation. The 1/e2 beam diameter was measured to be 0.80 mm (Nd:YAG) using the knife blade method. The laser intensity was modulated with the help of reflective power density neutral filters and measured with an optical power meter (Thorlabs PM100USB, power sensor S120VC). In the dual‐wavelength excitation experiments, the initial laser beams’ relative intensity has been tuned, taking into account the relative respective sensitizer absorbance, in order to constantly generate in the system the same number of excited sensitizers with green and red excitation light as a function of the optical density of the neutral filter used to modulate the fluence of the dual beam excitation (Figure S6, Supporting Information). The stray light of the laser was attenuated with notch filters in the detection line. All emission spectra were corrected for the optical response of the instrument. The samples were excited at 532 or 635 nm for time‐resolved upconversion measurements by modulating a Nd:YAG laser with a TTi TG5011 wavefunction generator with a duty cycle of 50%, in order to reach the steady‐state condition before monitoring the upconverted emission in intensity decay. All spectra were recorded using an N_2_‐cooled photomultiplier (Hamamatsu R5509‐73) that was coupled to a high‐speed amplifier (Hamamatsu C5594), a monochromator (ORIEL 74 100 Cornerstone 2601/4), and a PCI plug‐in multichannel scaler (ORTEC 9353 100 ps time digitizer/MCS) in photon‐counting acquisition mode.

## Conflict of Interest

The authors employer has been granted a patent that may protect the materials reported.

## Supporting information



Supporting Information

## Data Availability

The data that support the findings of this study are openly available in Zenodo at https://doi.org/[10.5281/zenodo], reference number 13709782.
