# Supplementary material for: Confinement‐Enhanced Multi‐Wavelength Photon Upconversion Based on Triplet–Triplet Annihilation in Nanostructured Glassy Polymers
Source: Adv Sci (Weinh). 2025 Feb 14;12(14):2415160. doi: 10.1002/advs.202415160 (PMC11984915; doi:10.1002/advs.202415160)
Supplement: Supplementary file 1 — Supporting Information [file ADVS-12-2415160-s001.docx]

**Supporting Information for**

**Confinement-enhanced multi-wavelength photon upconversion based on triplet-triplet annihilation in nanostructured glassy polymers**

*Xueqian Hu^1^, Luca Pollice^2^, Alessandra Ronchi^2^, Marco Roccanova^2^, Michele Mauri^2^, Davide Lardani^1^,* *Dimitri Vanhecke^1^, Angelo Monguzzi^2*^, and Christoph Weder^1*^*

^1^Adolphe Merkle Institute, University of Fribourg, Chemin des Verdiers 4, 1700 Fribourg, Switzerland

^2^Dipartimento di Scienza dei Materiali, Università degli Studi Milano-Bicocca, Via Roberto Cozzi 55, Milano 20125, Italy

**Table of Content**

1. Additional data…………………………………………………………………………………… Page S2

2. Modeling of confined-TTA in multi-wavelength upconverting nanostructured polymers……… Page S9

3. Upconversion quantum yield measurements……………………………………………………. Page S10

**
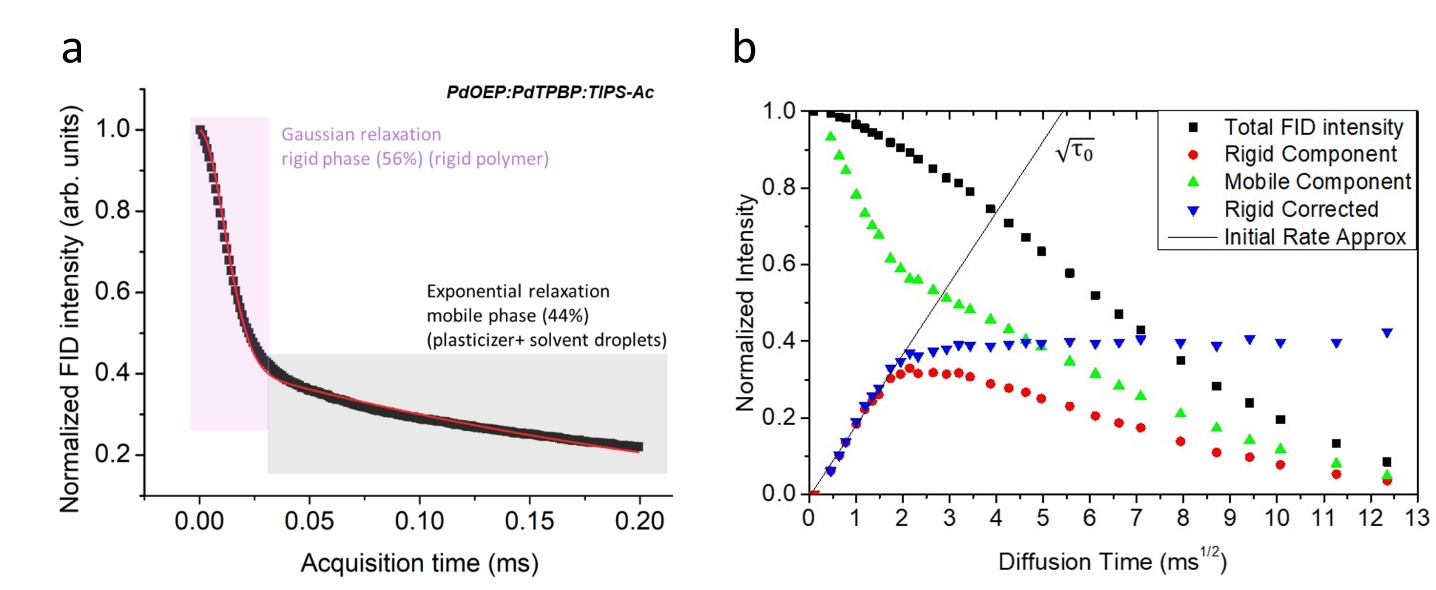
**

**Figure S1**. (a) Time-domain NMR FID of the nanophase-separated polymer containing three dyes PdOEP:PdTPBP:TIPS-Ac, acquired with MSE refocusing block at 303 K. The initial fast Gaussian relaxation characterizes the rigid phase, while the slower exponential relaxation is associated with the mobile phase. DPA and DPA:PdOEP lines are practically superimposed, indicating that the presence of the triplet sensitizer PdOEP does not affect the material structure. (b) Spin diffusion experiment performed on the upconverting (UC) material at 30 °C (303K). The total normalized intensity as a function of diffusion time is presented together with the contribution of the rigid and mobile components obtained by bimodal (gaussian + exponential) fitting of each separate experiment. To offset the T1 relaxation, the rigid fraction data were then corrected by pointwise normalization against the total signal intensity. On the corrected data, we applied the Initial Rate Approximation, obtaining a value for sqrt(tau_0) that is related to the average domain size of the mobile fraction.

**
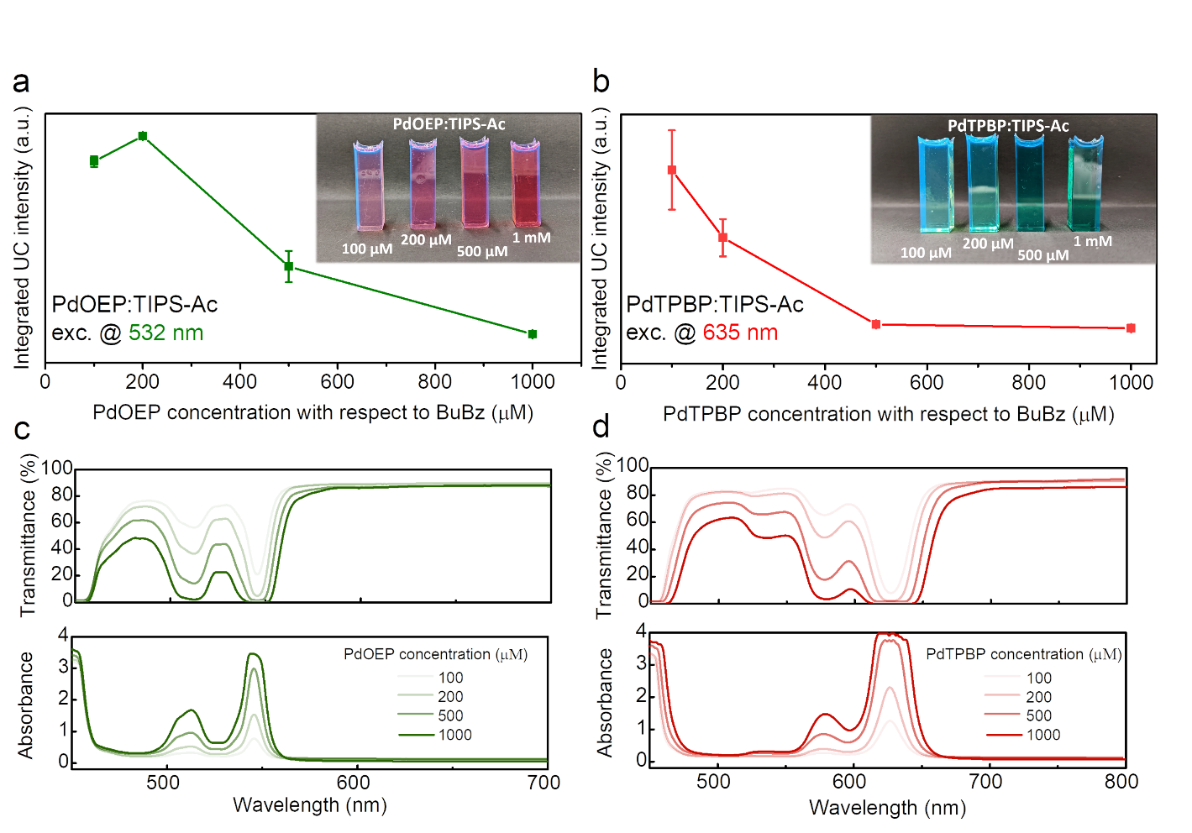
**

**Figure S2.** Integrated UC intensity (a,b) and transmission and absorption spectra (c,d) of upconverting nanostructured polymers containing PdOEP:TIPS-Ac (a,c) or PdTPBP:TIPS-Ac (b,d). The concentration of TIPS-Ac (20 mM with respect to BuBz) was kept constant, while the concentration of PdOEP (100 μM, 200 μM, 500 μM and 1 mM with respect to BuBz) and PdTPBP (100 μM, 200 μM, 500 μM and 1 mM with respect to BuBz) were systematically varied. The UC intensity was recorded with CW laser excitation at 532 nm (PdOEP:TIPS-Ac) or 635 nm (PdTPBP:TIPS-Ac). Insets in a,b show digital pictures of the samples under ambient light conditions.


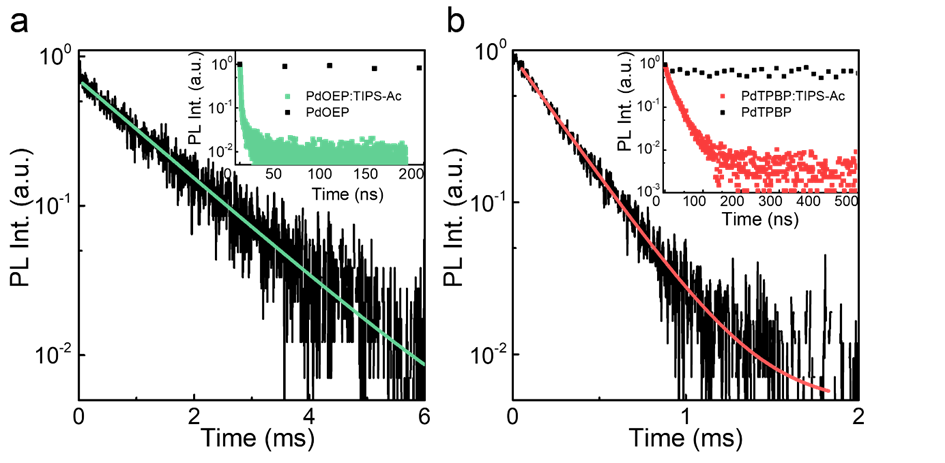


**Figure S3.** Phosphorescence intensity decay (black solid lines) recorded at 670 nm (a) and 800 nm (b) of nanostructured polymers containing only PdOEP (2×10^-5^ M) under pulsed excitation at 532 nm (a) and only PdTPBP (1×10^-5^ M) under pulsed excitation at 635 nm (b). The green and red lines are fits to the data with a single exponential decay function with a characteristic lifetime τ_ph1_ = 1.32 ms (a) and τ_ph2_ = 0.27 ms (b). The insets show the phosphorescence intensity decays of nanostructured polymers containing a) PdOEP (2×10^-5^ M) with (green squares) and without (black squares, TIPS-Ac (2×10^-3^ M) and b) PdTPBP (1×10^-5^ M) with (red squares) and without (black squares, TIPS-Ac (2×10^-3^ M).

**
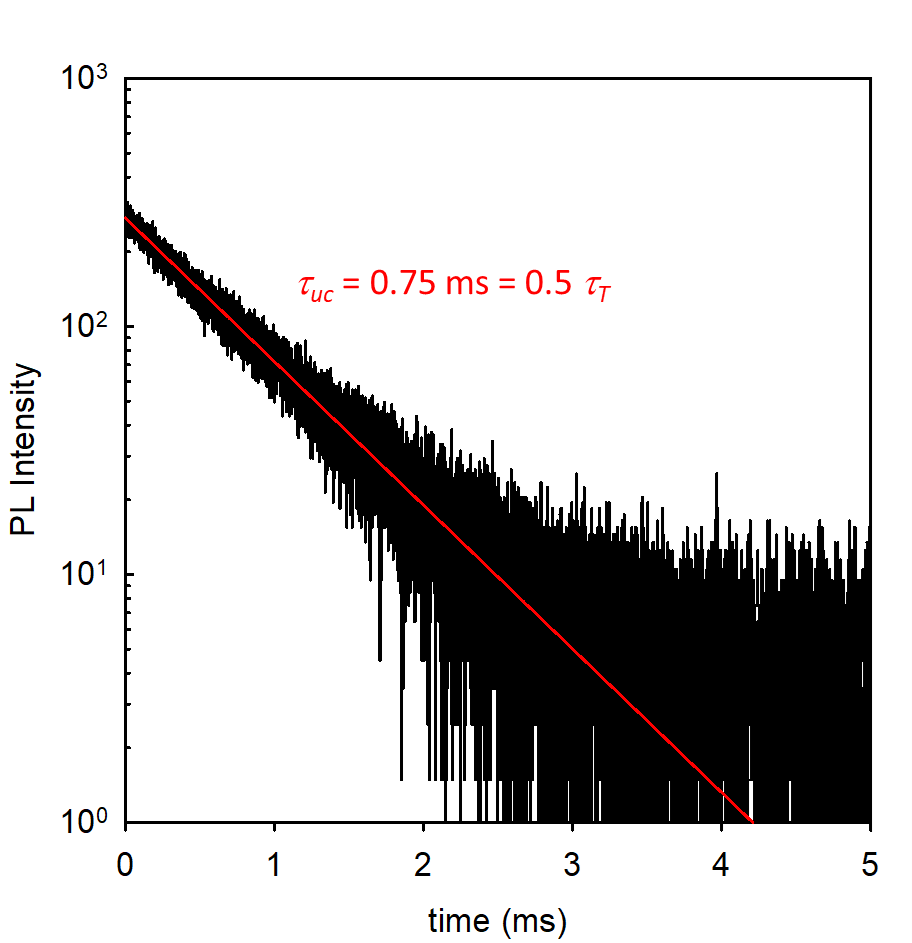
**

**Figure S4** UC emission intensity decay (black solid line) recorded at 475 nm in a BuBz solution containing only PdOEP (2×10^-5^ M) and TIPS-Ac (2×10^-3^ M) under pulsed excitation at 532 nm (0.05 mW cm^-2^). The solid line is the fit of signal decay with a single exponential decay function with characteristic decay time τ_uc_ = 0.75 ms, which is half of the TIPS-Ac triplet lifetime τ_T_ =1.5 ms. The spontaneous decay rate of the TIPS-Ac triplets is therefore k_T_ = (τ_T_)^-1^= 666 Hz.


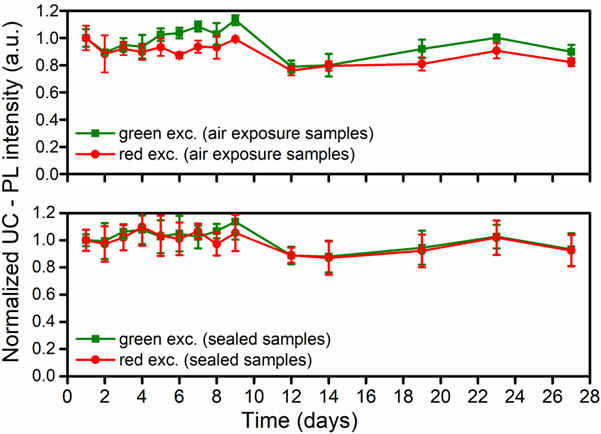


**Figure S5.** Normalized UC intensities of upconverting nanostructured polymers containing PdOEP (200 μM with respect to BuBz), PdTPBP (100 μM with respect to BuBz), and TIPS-Ac (20 mM with respect to BuBz), sealed in a cuvette (bottom) and released and exposed to air (top), under 532 nm or 635 nm CW laser excitation as a function of time. Each data point represents the average of data acquired for three different samples.


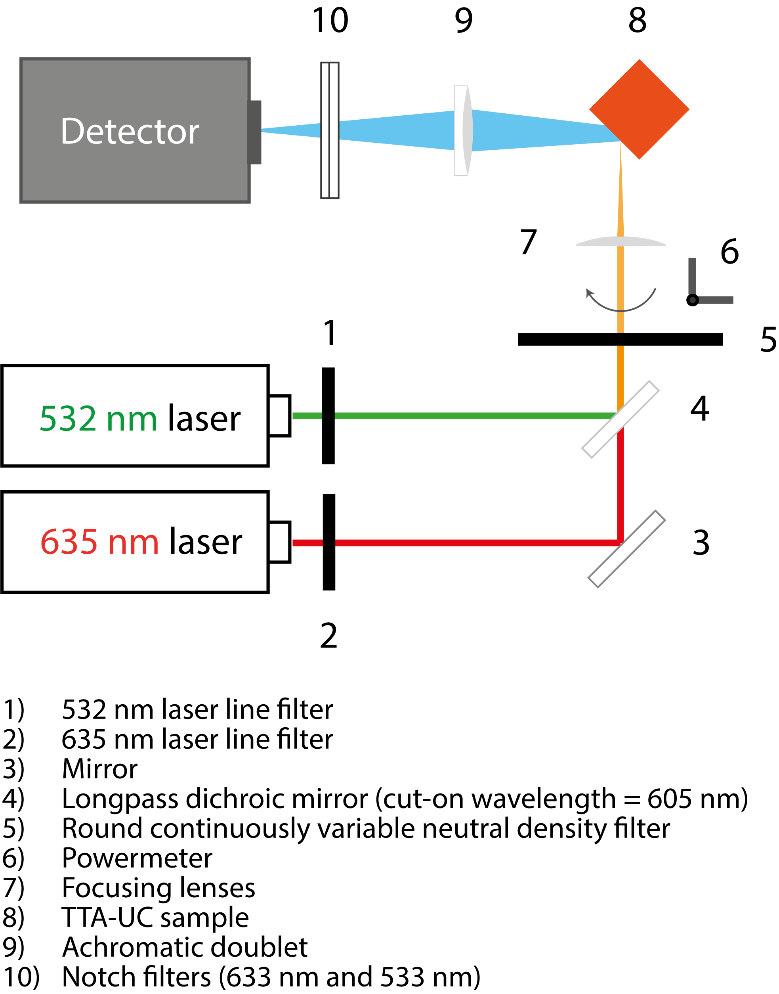


**Figure S6.** Schematic of the optical setup used for steady-state upconversion measurements. The initial beams’ relative intensity has been tuned, taking into account the relative respective sensitizer absorptance in order to constantly generate in the system the same amount of excited sensitizers with green and red excitation light as a function of the OD of the neutral filter used to modulate the fluence of the dual beam excitation.

**Table S1.** Parameters employed to calculate the TTA yield as $\phi_{TTA}=\frac{k_{TTA}}{k_{TTA}+k_{T}}$ where *k_t_* = 666 Hz is the spontaneous decay rate pf the TIPS-Ac annihilator triplet in BuBz (Fig. S4). The UC emission decay rate $k_{TTA}$ has been calculated as the reciprocal of the time $\tau_{uc}$ at which the UC emis­sion intensity is reduced to 1/*e* of its initial value fitting the data with a multiexponential decay function (Figure 3). We used a multiexponential decay function because in the nanostructured system is not possible to apply the same analytical expression to describe the decay kinetic of TTA-based delayed fluorescence in bulk systems where an infinite volume is available for diffusing triplets.^[3] [4]^

| **Sample** | **I_exc_** | **τ_uc_ [ms]** | **k_TTA_ [Hz]** | **φ_TTA_** |
| --- | --- | --- | --- | --- |
| PdOEP:TIPS-Ac  exc 532 nm | 20.0 W cm^-2^ | 0.029 | 34483 | 0.98 |
|  | 5.1 W cm^-2^ | 0.036 | 27778 | 0.97 |
|  | 0.2 Wcm^-2^ | 0.032 | 31250 | 0.98 |
|  |  |  |  |  |
| PdTPBP:TIPS-Ac  exc 635 nm | 24.35 W cm^-2^ | 0.031 | 32258 | 0.97 |
|  | 6.2 W cm^-2^ | 0.021 | 47619 | 0.98 |
|  | 0.1 Wcm^-2^ | 0.030 | 33334 | 0.98 |
|  |  |  |  |  |
| PdOEP:PdTPBP:TIPS-Ac  exc 532 nm | 20.0 W cm^-2^ | 0.031 | 32258 | 0.98 |
|  | 5.1 W cm^-2^ | 0.037 | 27027 | 0.98 |
|  | 0.2 W cm^-2^ | 0.034 | 29411 | 0.98 |
|  |  |  |  |  |
| PdOEP:PdTPBP:TIPS-Ac  exc 635 nm | 24.3 W cm^-2^ | 0.031 | 32258 | 0.98 |
|  | 6.2 W cm^-2^ | 0.033 | 30303 | 0.98 |
|  | 0.2 Wcm^-2^ | 0.036 | 27777 | 0.98 |


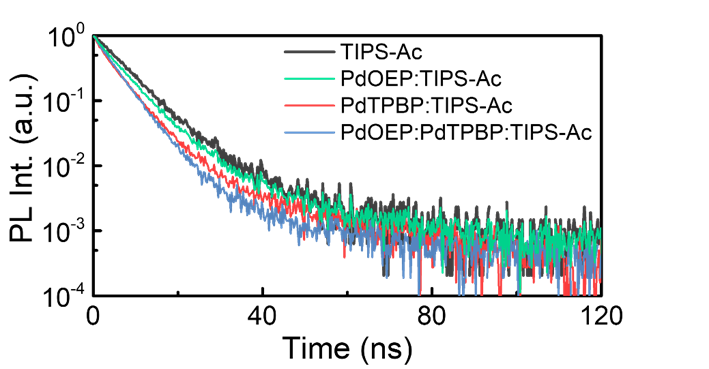


**Figure S7.** Time-resolved PL decay spectra of nanostructured polymers containing only TIPS-Ac (2×10^-3^ M, black line), PdOEP and TIPS-Ac (2×10^-5^ M and 2×10^-3^ M, green line), PdTPBP and TIPS-Ac (1×10^-5^ M and 2×10^-3^ M, red line), and PdOEP, PdTPBP, and TIPS-Ac (2×10^-5^ M, 1×10^-5^ M, and 2×10^-3^ M, blue line), recorded at 450 nm under pulsed laser excitation at 405 nm.





**Figure S8.** The size and distribution of nanodroplets in a nanostructured multi-wavelength upconverting polymer, data collected and calculated based on three different areas from SEM images using Fiji (ImageJ) software. Assuming spherical nanodroplets for simplicity, the Gaussian fit reveals an average nanodroplet mean diameter of around 22 nm.


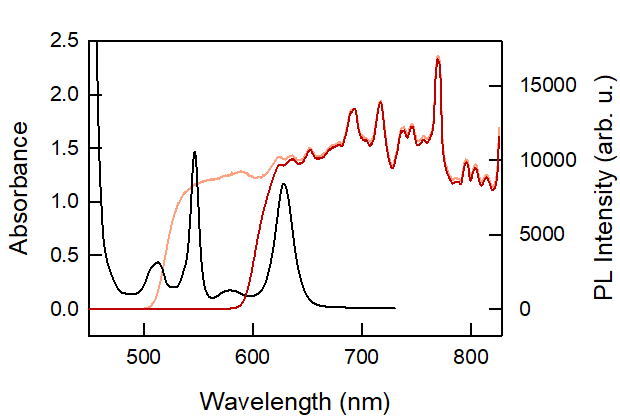


**Figure S9.** UV-vis absorption spectrum of the dual sensitizer nanostructured glassy polymer (black line) and broadband excitation light using a long pass filter at 520 nm (yellow line) and 600 nm (red line.)


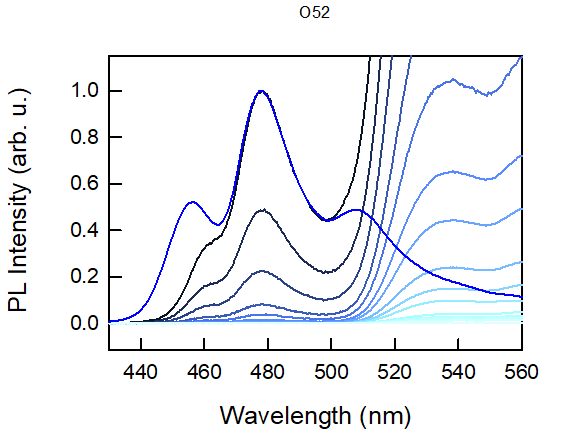


**Figure S10.** UC PL emission spectrum of the dual sensitizer nanostructured glassy polymer as a function of the excitation intensity under broadband light filtered using a 520 nm long pass optical filter. The dark blue curve with three maxima between 440 nm and 520 nm is the spectrum taken by directly exciting the emitter without filtering the lamp.


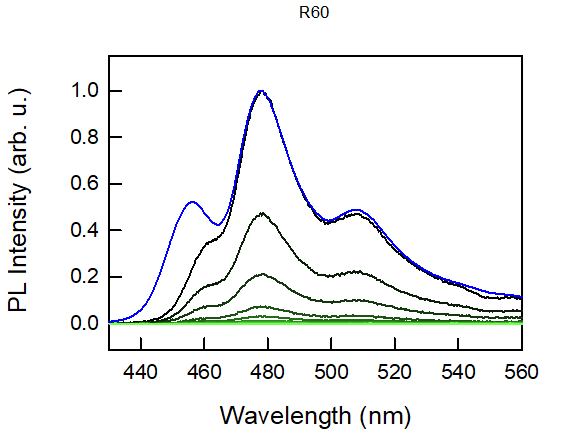


**Figure S11.** UC PL emission spectrum of the dual sensitizer nanostructured glassy polymer as a function of the excitation intensity under broadband light filtered using a 600 nm long pass optical filter. The dark blue curve with three maxima between 440 nm and 520 nm is the spectrum taken by directly exciting the emitter without filtering the lamp.

**Table S2.** Absorption properties of the ideal solar device with bandgap 520 nm, coupled to a UC layer made of dual-sensitizer nanostructure polymer under exposure to sunlight in AM1.5 condition. The system absorptance increment is calculated considering the measured sample absorption in the triplet sensitizers' green and red bands. The charge generation increment in the solar device is calculated considering the maximum UC yields measured, which can be obtained in a real device by concentrating the residual non-absorbed solar emission with a lens. A back reflector drives all the upconverted blue photons into the solar device to be absorbed. We consider a constant internal photon-to-charge conversion yield.

| **Sample** | **Absorbed photons** | **Absorptance**  **increment** | **Charge generation increment** |
| --- | --- | --- | --- |
| Solar device (bandgap 520 nm) | 5.68e+16 | +0.38 | +0.07 |
| PdOEP green band  (peak 542 nm) | 8.16e+15 |  |  |
| PdTPBP red band  (peak 635 nm) | 1.36e+16 |  |  |
|  |  |  |  |

**2.** **Modeling of confined-TTA in multi-wavelength upconverting nanostructured polymers**

We developed a model to describe the different behavior of the UC yield as a function of the excitation intensity in nanostructured upconverting polymers with two different sensitizers embedded in the liquid phase when the system is under a single laser excitation or a simultaneous double-laser excitation.

The UC yield at a given excitation intensity $I_{exc}$ [$\mathrm{ph}\mathrm{cm}^{-2}s^{-1}$] is set in the first place by the overall triplet exciton density $T_{E}$ [$\mathrm{cm}^{-3}$] generated in the polymer during the emitter triplet lifetime $\tau_{T}$=1.55 ms, given by

$T_{E}=\alpha\phi_{ET}I_{exc}\tau_{T}\rho$ Eq. S1

where $\alpha$ [${cm}^{-1}$] is the absorption coefficient at the single excitation wavelength, $\phi_{ET}$ is TTET efficiency and $\rho=10\%$ is the fraction of liquid phase in the overall polymer that embeds the upconverting dyes.

Given the homogenous nature of the polymerization process, the upconverting liquid nanodroplets assumed as spheres of radius $r$ for simplicity, are distributed in size according to a log-normal distribution $L\left( r \right)$

$L\left( r \right)=\frac{1}{\sqrt{2\pi}\sigma r}e^{-\frac{{(\ln\left( r \right)-\mu)}^{2}}{2\sigma^{2}}}$ Eq. S2

where $\mu$and $\sigma$ are the mean value and the standard deviation of $\ln\left( r \right)$ respectively.^[1]^ According to time-domain NMR measurement, the liquid droplets’ mean radius was 7.5 nm, corresponding to $\mu=1.625$ and $\sigma=0.88$.

As already demonstrated in a previous work^[2]^, the photophysical behavior of the nanostructured polymer is described by an effective radius-weighted log-normal distribution $L^{'}\left( r \right)=L\left( r \right)\times r$ to properly account for the different absorbing abilities of the nanodroplets according to their size.

Therefore, the mean number of triplet excitons created in a single nanodroplet of volume V is $n= T_{E}\times$ V. The TTA is activated in a single nanodroplet when at least two triplets are created simultaneously in the same volume, so the nanodroplets are activated at different powers, according to size-dependent binomial statistics of excitation energy distribution.

Therefore, in the case of a single laser excitation, the UC yield is set by the cumulative probability $P_{n\geq2}$ to have 𝑛 ≥ 2 triplet excitons per nanodroplet expressed by the Poisson distribution. The normalized UC yield can be approximated as

$\phi_{UC}^{single}=\sum_{r} (P_{n\geq2,r}\times L^{'}(r))$ Eq. S3

so the cumulative probability of having at least two excitons in the same nanodroplet of size *r* has to be multiplied by the weight of that specific size according to the effective log-normal distribution.

In the case of the simultaneous double-laser excitation, the situation is different. Now the nanodroplet is still activated when at least two excitons are created simultaneously, but it is enough that at least one triplet exciton is created by sensitizer 1 ($P_{n\geq1}^{1}$) and the other by sensitizer 2 ($P_{n\geq1}^{2}$) to activate the nanodroplet.

Therefore, since $P_{n\geq1}^{1}$ does not influence $P_{n\geq1}^{2}$, in this case, the UC yield is approximated as

$\phi_{UC}^{double}=\sum_{r} (P_{n\geq1,r}^{1}{\times P}_{n\geq1,r}^{2}\times L^{'}(r))$ Eq. S4

**3.** **Upconversion quantum yield measurements.**

The up-conversion luminescence quantum efficiency ${QY}_{uc}$ of polymeric up-converters under 532 nm excitation was determined relative to the DPA-PtOEP UC pair in deaerated tetrahydrofuran ([DPA] = 10 mM, [PtOEP] = 100 μM) used as secondary standard, according to the following equation

${QY}_{uc} = {QY}_{std}\left( \frac{A_{std}}{A_{uc}} \right)\left( \frac{I_{uc}}{I_{std}} \right)\left( \frac{P_{std}}{P_{uc}} \right)\left( \frac{\eta_{uc}}{\eta_{std}} \right)^{2}$ Eq. S2

where ${QY}_{uc}$, *A_uc_*, *I_uc,_ P_uc_* and *η_uc_* represent the quantum yield, absorptance at the excitation intensity integrated photoluminescence spectral profile, excitation power density, and refractive index of the medium. The corresponding terms for the subscript “*std*” are for the reference quantum counter of DPA-PtOEP pair in deaerated tetrahydrofuran at the identical excitation wavelength. The theoretical maximum UC quantum efficiency is 0.50 since the TTA-UC process uses 2 low energy photons to produce 1 high energy photon. The UC quantum yield of the secondary standard is 0.26 at its maximum.^[5]^ The recorded spectra have been corrected for the setup optical response.

The secondary standard solution is freshly prepared and characterized before each run of measurements. In such a way, we can avoid to perform the spectral correction for all the UC samples. It should be noted that in the high excitation power density limit the ${QY}_{uc}$ does not depend on the excitation intensity anymore and reaches its constant maximum value. The refraction index of tetrahydrofuran is 1.41 at 293 K. The refraction index for the polymer series analysed varies in the range from 1.45 to 1.47, as reported in the literature, giving a negligible correction to the measured ${QY}_{uc}.$

For the the dual-sensitizer nanostructured polymer we measured a maximum UC yield of 24±4% with an absorbed photon flux of 5×10^19^ ph cm^-2^ s^-1^ under excitation at 532 nm (Figure 3c).

For the UC pair excited at 635 nm, we employed as reference de-oxygenated standard solution of the sensitizer PdTPBP, which phosphorescence quantum yield of 0.06 has been measured relative to that one of PtOEP under the same excitation at 405 nm.^[6]^ For the the dual-sensitizer nanostructured polymer we measured a maximum UC yield of 16±3% with an absorbed photon flux of 10^19^ ph cm^-2^ s^-1^ at 635 nm (Figure 3d).

**Supplementary references**

[1] J. Söderlund, L. B. Kiss, G. A. Niklasson, C. G. Granqvist, *Phys. Rev. Lett.* **1998**, *80*, 2386.

[2] F. Saenz, A. Ronchi, M. Mauri, D. Kiebala, A. Monguzzi, C. Weder, *ACS Appl. Mater. Interfaces* **2021**, *13*, 43314.

[3] Monguzzi A., et al. *J. Phys. Chem. Lett*. **2016**, 7, 2779−2785

[4] Meinardi F. et al., *Nano Lett*. **2019**, 19, 2169−2177

[5] Monguzzi, A., Tubino, R., Hoseinkhani, S., Campione, M. & Meinardi, F. Low power, non-coherent sensitized photon up-conversion: modelling and perspectives. *Phys. Chem. Chem. Phys.* **14**, 4322-4332 (2012)

[6] A. K. Bansal, W. Holzer, A. Penzkofer, and T. Tsuboi, *Chem. Phys*. **2006**, **330**, 118.
